# Training Compliance Control Yields Improvements in Drawing as a Function of Beery Scores

**DOI:** 10.1371/journal.pone.0092464

**Published:** 2014-03-20

**Authors:** Winona Snapp-Childs, Ian Flatters, Aaron Fath, Mark Mon-Williams, Geoffrey P. Bingham

**Affiliations:** 1 Department of Psychological & Brain Sciences, Indiana University, Bloomington, Indiana, United States of America; 2 Institute of Psychological Sciences, University of Leeds, Leeds, United Kingdom; 3 School of Mechanical Engineering, University of Leeds, Leeds, United Kingdom; Goldsmiths, University of London, United Kingdom

## Abstract

Many children have difficulty producing movements well enough to improve in sensori-motor learning. Previously, we developed a training method that supports active movement generation to allow improvement at a 3D tracing task requiring good compliance control. Here, we tested 7–8 year old children from several 2^nd^ grade classrooms to determine whether 3D tracing performance could be predicted using the Beery VMI. We also examined whether 3D tracing training lead to improvements in drawing. Baseline testing included Beery, a drawing task on a tablet computer, and 3D tracing. We found that baseline performance in 3D tracing and drawing co-varied with the visual perception (VP) component of the Beery. Differences in 3D tracing between children scoring low versus high on the Beery VP replicated differences previously found between children with and without motor impairments, as did post-training performance that eliminated these differences. Drawing improved as a result of training in the 3D tracing task. The training method improved drawing and reduced differences predicted by Beery scores.

## Introduction

Previously we created a method for training children to develop better compliance control to enable them to improve their manual actions, including drawing and or handwriting [1]. The training method was developed to overcome a problem faced by children in sensori-motor learning, and thus, to allow them to learn effectively. Also, because it is automated, the training method does not, in principle, require the presence of a trainer. Here, we expand on this work by examining learning in a broader context and whether training has a wider benefit (i.e. transfer of learning). Previously, we tested children diagnosed as having Developmental Coordination Disorder (DCD). Developmental Coordination Disorder is understood to be a perceptuo-motor disorder [2] where children exhibit poor gross motor control, poor fine motor control, or both [3]. Now we test school children who exhibit a range of fine motor coordination abilities. We also test whether the training transfers to improved drawing ability.

### Active prospective control is required for effective sensorimotor learning

Sensori-motor learning has been characterized as involving two stages [4]. The first stage is to produce a movement that approximates that represented by the skill. Subsequently, practice of the qualitatively correct, but quantitatively poor movement yields quantitative improvement and development of the skill. In general, it can be difficult for children to produce the initial approximation required for practice to yield effective sensori-motor learning. This is clearly seen in very early childhood and infancy (i.e. [5]) but is also evident later in childhood when skilled and targeted actions are considered i.e. the development of throwing (see [6]–[7]). So, then, the issue becomes how to aid or train children to produce qualitatively appropriate movements. A traditional approach used by teachers and movement therapists to overcome this problem is to model desired movement skills with the hope that the learner will approximate some form of the required skill and then improve with practice. Accordingly, the expert will move the limbs of the learner through a desired form of movement (called “active assist”). Similar robotic approaches to therapy have been developed to move the passive limbs of the learner through the to-be-acquired movements; in effect, these robotic approaches “replace” the therapist (for reviews, see [8]–[9]). Generally, however, passive robotic approaches to therapy for adults have not been found to be effective [10]–[12]. Moreover, passive training of movements in healthy adult populations appears not to lead to robust learning (for examples, see [13]–[14]).

It is not entirely clear why passive training is ineffective. However, there are several plausible explanations. One possibility is that because the muscles are inactive, the sensory support for control of the muscles (e.g. muscle spindles and golgi receptors in tendon) are also inactive. This is consistent with work showing that 1) kinesthesis is significantly better in the context of actively controlled posture and movement [15]–[17], and 2) somatosensation is intrinsic to the control of joint posture and movement [18]. In the performance of actions, the current state of the motor apparatus must be perceived relative to the constraints imposed on the action by the environment to allow effective motor control — effective proprioception is essential for this. An alternative explanation is that the absence of prospective control that renders passive movements ineffective for sensori-motor learning; prospective control is the guidance of movements based on future-specific information [19]–[25]. Snapp-Childs, Casserly, Mon-Williams, and Bingham [26] tested these alternatives by comparing the performance of young, healthy adults who were trained with either an active or a passive version of a 3D tracing task; the passive task was a haptic tracking task in which participants grasped a stylus moved by a PHANTOM Omni and moved their limb to track the movements of the stylus. The musculature was active, but the generation of the movements did not entail prospective control and active generation of movement trajectories that anticipated the path of movement along the target path. The goal of that study was to investigate whether effective motor learning would be allowed by passive control (i.e. control lacking an active prospective perceptual component). The results showed a clear advantage of active training over the passive version (where, again, the passive training was passive in that there was a lack of prospective control and not the quiescence of the musculature).

### Variability, stiffness, and compliance control

One of the hallmarks of typical motor development is increased movement consistency with age or experience. Younger children have more variable motor skills relative to older children and it appears to be consistent across a wide variety of tasks [27]–[29]. For example, Snapp-Childs and Bingham [27] showed that younger children (4-years old) produced more variable obstacle crossing behaviors when compared to older children (6-years old) and adults. Likewise, Deustch and Newell [28] showed that the constancy of continuous isometric force production (with the index finger) improved with increasing age (from 6–10 years of age). It is not entirely understood how children manage to increase their consistency when performing whole body, dynamic actions such as in the case of crossing obstacles while walking. However, for upper limb movements and control, a common strategy for performing in the face of high variability is to adopt high muscular stiffness (which is the inverse of compliance) [30]–[31]. High stiffness usually reduces the effect of perturbations, but is exhausting to maintain so low stiffness is typically used later in the learning process once variability decreases (for a review, see [30]). With recent advances in robotics, researchers have been able to exploit such features of motor control. For example, Ben-Pazi and collaborators used virtual reality technology (PHANTOM Omni) to externally impose increased viscosity and inertia on children's movements when performing a handwriting task [32]. They showed imposing these constraints yielded improvements in control resulting in improved handwriting performance.

Previously, we created and tested a method for training children to develop better compliance control (compliance is the inverse of stiffness) to enable them to improve their manual actions [1]. The rationale was that school aged children (6+ years old) can generate task-appropriate amounts of force but their inconsistent performance levels interferes with proper stiffness control. To address this, we created a 3D tracing task that employed computer graphics and force feedback haptic virtual reality technology (PHANTOM Omni) [1]. The technology allowed us to vary task parameters in a way that enabled children to succeed. Moreover, the training itself was structured so that early in training the precision required to do the task was low. Training progressed from lower to higher precision (and limb compliance) requirements as the children achieved task mastery. High self-efficacy was maintained in this way. The end result was that the training was successful and all of the children greatly improved.

### Present study

One of the limitations of the Snapp-Childs et al. study [1] was that it did not examine whether the training had a wider benefit. Here, we examine learning in a broader context and whether training has a wider benefit. Studies involving school-aged children often test the participants using a standardized test (e.g. Movement ABC and others in the UK, Beery VMI or Peabody and others in the US) and use the test scores as part of the inclusion criteria e.g. [33]–[34]. Here, we also tested children with a standardized test (Beery VMI) but did not exclude children on the basis of this test. Instead, we used it as information about fine motor coordination skill. We selected the Beery VMI because it tests fine motor control tasks related directly to handwriting [35]–[37] (and the target of our method of training is improved handwriting) and also because the Beery is a popular assessment choice that is used widely by clinicians and researchers in the US [34], [38]. First, we directly investigated the relation between performance by school children (second graders, 7–8 year olds in a local public primary school) on the Beery VMI and on a 3D tracing task. Second, we investigated whether our training method and task yielded improvements in performance of a drawing task. We also examined the relation of performance on the Beery VMI to the performance in the drawing task. An advantage of this approach is that we were able to use continuous (regression) analysis in addition to discrete or categorical analysis of variance to relate the continuous variations in performance in each of the tasks, namely, Beery, 3D tracing, and drawing.

The first question was whether Beery scores would predict baseline performance in our 3D tracing task and then, if so, whether the training regime would yield a similar improvement in performance in posttest as observed in the previous study, so as to eliminate the (Beery-predicted) performance differences observed in baseline. We predicted that baseline performance of the 3D tracing task should vary as a function of the Beery scores. The 3D tracing task has a strong visuo-motor component, so we predicted that it is the performance on the VMI subtest that should best predict 3D tracing performance. Second, we predicted that training on the 3D tracing task would yield improvements that eliminate the differences in performance observed in baseline as a function of Beery scores.

The training in the 3D tracing task is intended to yield improvements in handwriting and drawing performance, enabled by improved compliance control. So, the second question we examined was whether training on the 3D tracing task would yield improvements in performance of the drawing task and if so, would these vary as a function of scores on the Beery inventory. We predicted that training on the 3D tracing task should yield improvements in the drawing task. We also predicted that, before training, performance on the drawing task should vary as a function of the Beery scores. Again, because this task has a strong visuo-motor component, we predicted that it would be the VMI subtest that would best predict drawing performance. Unlike the 3D tracing task, we predicted that the Beery scores would still predict drawing performance after training, though we expected the relationships to be maintained because we anticipated relatively equal improvement.

## Methods

### Participants

Twenty-eight children, 7- and 8-years old, were recruited from four 2^nd^ grade classrooms in a local elementary school. All children, save one, were right-handed. Several children yielded incomplete data sets because they were absent from some of the testing sessions or because they failed to keep the stylus in contact with the touch screen during the handwriting tests. So, only 23 children yielded complete data included in the analyses. Of these children, 8 were female and 15 were male.

### Ethics Statement

This study was approved by the Indiana University Institutional Review Board. The children participated with informed assent with (written) informed consent from their parents/guardians.

### Procedure and Apparatus

Before testing began, the parents/guardians evaluated their child using the Developmental Coordination Disorder Questionnaire (DCD-Q '07) [39]; these were completed by the parents/guardians at home. The children were tested for all sessions at their school. During the first testing session, all participants were evaluated by a trained clinical psychology doctoral student using the Beery-Buktenica Developmental Test of Visual-Motor Integration (Beery). The participants also completed a drawing task and a 3D tracing task. In a number of subsequent sessions, participants completed a customized sensori-motor training program. After training, participants repeated the assessments of drawing and handwriting and 3D tracing.

#### Beery

There are three components of the Beery: tests of 1) visual-motor integration (VMI), 2) visual-perception (VP), and 3) motor coordination (MC). The Beery VMI consists of 24 items (geometric forms) that are to be copied with pencil and paper. The VP and MC use the same geometric forms as the VMI, but the goals are different. In the VP, the goal is to choose one form, from a few slightly different alternatives, that is exactly the same as the stimulus. The alternatives can be very slightly different in form or size. In the MC, the goal is to trace inside (double) lines that define the stimulus forms.

#### Drawing

In the drawing test, participants were seated at a table in front of a tablet PC (Toshiba Portégé M750 tablet PC, screen size 163 mm by 260 mm, using CKAT software to manage stimulus presentation, user interface, and data collection as described by Culmer and collaborators [40]). The task was to view a form, then to copy (not trace) the form on the computer screen using a handheld stylus in the dominant hand. When a trial started, the upper half of the screen contained a black rectangular frame (12×6.5 cm for Paths A, B, and C; 10×10 cm for Path D) around a black line form and the lower half of the screen contained a green rectangle of equal dimensions to the black frame.

**Figure 1:**
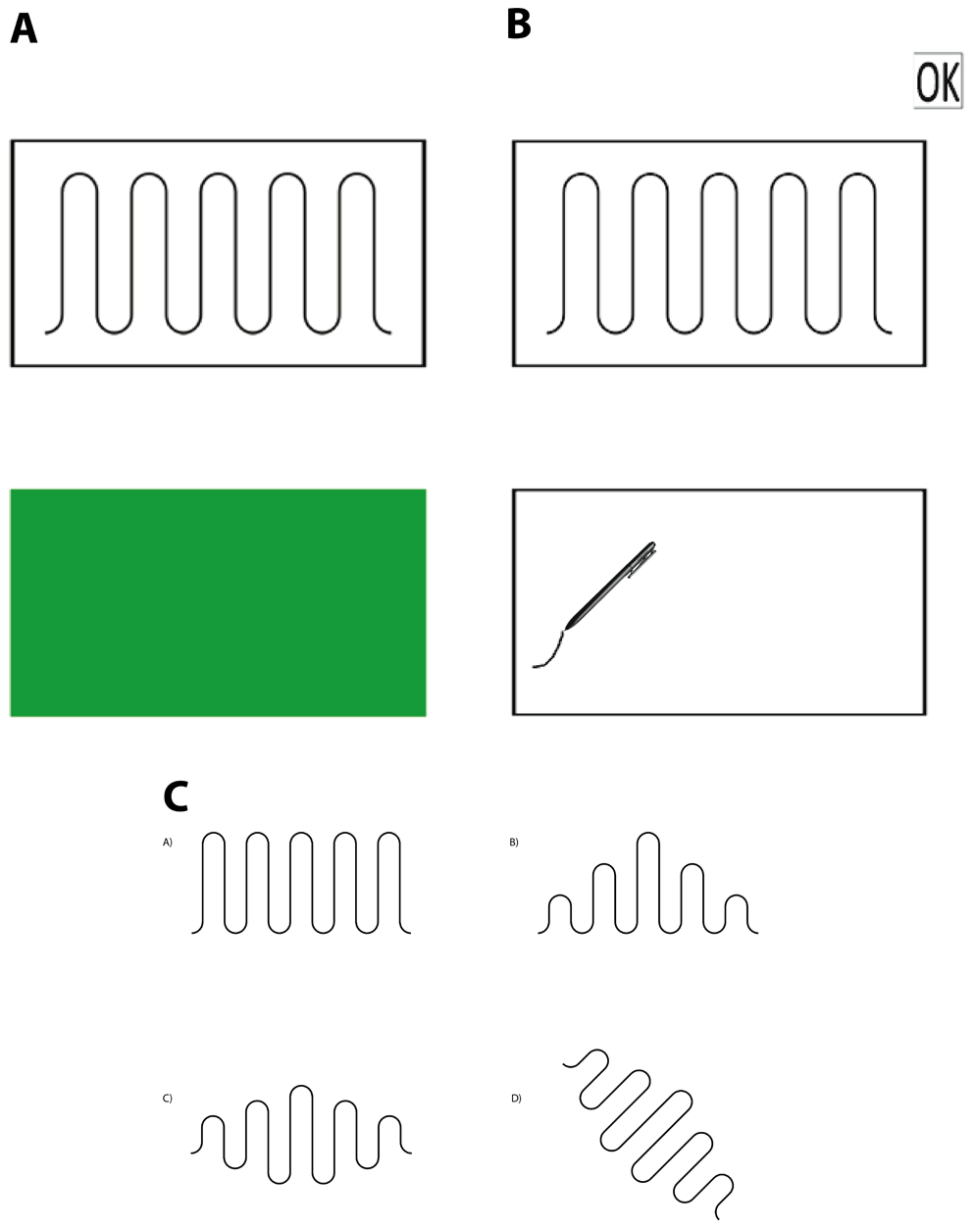
Illustrations of the drawing task and the four target forms copied by participants.

Participants looked at the form inside the black frame, then they placed the hand held stylus on the green rectangle at the location where they would start copying the form (see Figure 1a). Once the stylus was inside the rectangle for 200 ms, the green rectangle disappeared and was replaced with a white rectangle (same color as the background) with a black border around it – similar to the rectangle in the upper portion of the screen containing the form to be copied. Once participants began to draw the form, an “OK” button appeared in the upper right-hand corner of the screen (see Figure 1b). When participants finished copying the form, they tapped this button with the stylus, completing the current trial and beginning the next one.

Participants performed three practice trials (a horizontal line segment, one cycle of a sine wave, and a circle) that were not analyzed, to become familiar with the task and interface. Then, participants completed three repetitions of each of four forms (shown in Figure 1c), for a total of twelve trials. The paths had equal maximum height, maximum width, and wavelength, but varied in amplitude and orientation. Each crest and trough of Form A had equal amplitudes, resulting in waves with heights of 46 mm. Amplitude sequentially varied over the crests of Form B, but not the troughs, resulting in waves with heights of 16, 31, and 46 mm. Amplitude sequentially varied over both the crests and troughs of Form C. Thus, individual waves had sides of unequal height. The distances between neighboring maxima and minima were 16, 23.5, 31, 38.5, and 46 mm. Form D was the result of a 45° clockwise rotation of Form C. The variations in amplitude resulted in different path lengths. Form A was 527 mm long, Form B was 365 mm, and Form C and D were 392 mm.

#### 3D tracing

The 3D tracing task was similar to that described and used in two previous studies [1], [26]. In this task, participants performed variations of the same three-dimensional tracing task while seated at a table. The basic task was to push a brightly colored fish along a visible curved path viewed on a computer screen from a starting location (a plain square) to a finishing point (a checkered square) while racing a competitor fish. The participants grasped a stylus that was attached to a desktop force feedback haptic virtual reality device (PHANTOM Omni from Sensable Technologies) and used the stylus to feel the wire path and push the fish.

The PHANTOM is an impedance control device where the user moves the stylus and the device reacts with a force if a virtual object is encountered; the PHANTOM, thus, has displacement as an input and force as an output. The mass and friction of the PHANTOM has been made small by careful mechanical design. In this experiment, participants could “feel” the 3D path once they encountered it; phenomenologically, it was as if the stylus was “magnetically attracted” to the path. The force pulling the stylus was modeled as a virtual spring where the stiffness of the spring could be altered. The spring had a virtual length of ≈0.5 cm from the center of the path so the force dropped to zero if the stylus moved >0.5 cm from the path. The spring stiffness (and consequently the level of “attraction” or support) was parametrically varied to alter task difficulty. The forces pulling the stylus towards the spring were set at eight different levels corresponding to forces of approximately 3.04N, 2.77N, 2.02N, 1.08N, 0.83N, 0.57N, 0.35N and 0.13N.

**Figure 2:**
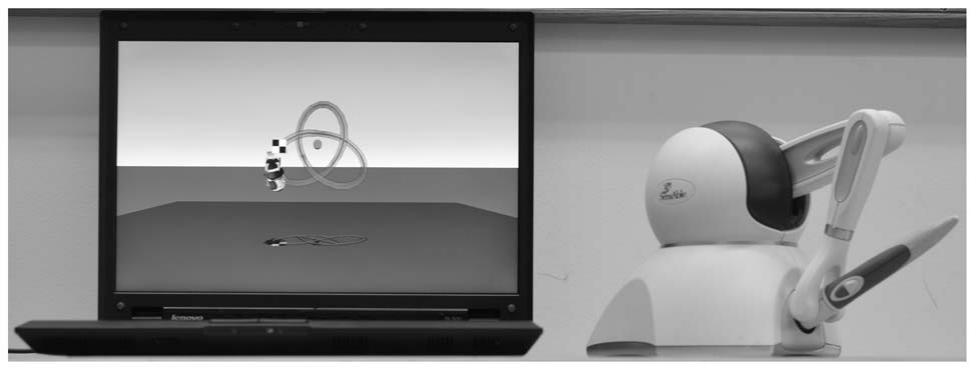
The PHANTOM Omni with the display. The display shows the path used in baseline and posttest trials.

The curved paths were similar to a toy, commonly found in pediatrician waiting rooms, consisting of brightly colored curved ‘roller coaster’ wires with beads on them that can be pushed along the wires by a child. However, doing this using a stylus to push the beads along the wire would be, and is for our task, very difficult. Hence, for our task, the path ‘magnetically attracted’ the stylus to hold it on the path. The ‘magnetic strength’ was parametrically varied, as described above, to alter task difficulty. At Baseline and Post-Training, participants attempted two trials at each of eight levels of support (‘magnetic attraction’), on the path pictured in Figure 2a, while racing a competitor fish that took 20 s to travel the path from start to finish. From earlier studies, it was clear that most children would spend a very long time to complete a path and would become very frustrated with the lack of progress. So, each trial was terminated if a child could not complete more than one half of the path within 60 s.

The training program consisted of up to five 20-minute training sessions that were separated by at least one week. During the training sessions, participants performed a series of 3D tracing tasks that were very similar to those in the Baseline/Post-Training sessions, but varied in length, curvature, and torsion (see Figure 2a, b, c). During training, participants raced against two different competitors; one competitor completed the path in 30 s while the other completed the path in 10 s. The first training session started with the highest level of support (‘magnetic attraction’), slowest competitor, and shortest path. The goal of the training was to allow the children to progress at their own pace through the different combinations of levels of attraction, paths, and competitors, so we used a “two-wins-in-a-row” rule to determine when the children progressed. After the participant “beat” the slowest competitor two times-in-a-row they progressed to the faster competitor. Once the participant beat both competitors they then moved to the next longest path with slowest competitor. After all paths and competitors were “beaten”, the level of support was decreased and the participant re-started with the shortest path and slowest competitor.

### Data analysis

#### DCD-Q

The scores reported here are the raw scores from the DCD-Q.

#### Beery

The scores reported here are the norm-referenced percentile scores for the VMI, VP and MC.

#### Drawing

**Figure 3:**
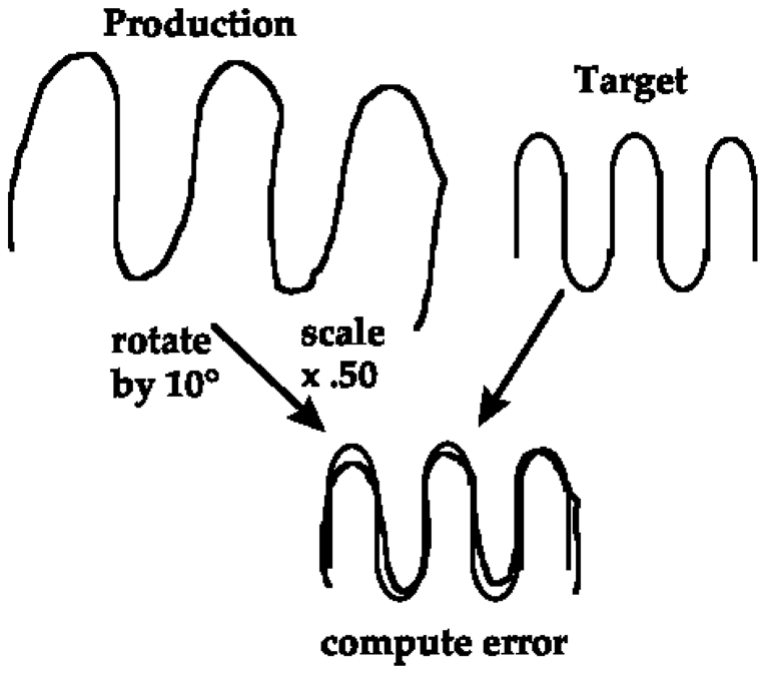
Illustration of the computer based method used to analyze drawn reproductions of the target forms.

The two-dimensional coordinates of the stylus were recorded at 120 Hz. These data were filtered using a dual-pass, second-order Butterworth filter with a 10 Hz cut-off frequency. We calculated three variables for each of the forms that participants produced: the scale factor, rotation, and shape accuracy [41]. See Figure 3 for illustration. To do this, we used a technique called ‘point-set registration’. In this technique, point-sets were generated for the participant-generated paths and reference paths by resampling the spatial coordinates, using linear interpolation, at a resolution of 1 mm. We then used a robust point-registration method [41]–[42] to determine the transformation that makes the participant-generated path most closely match the reference path. The transformation consisted of translation, rotation and isotropic scaling components. Scale factor is the isotropic scaling component of the transformation; that is, scale factor is how much growing or shrinking is required to make the participant-generated paths best match the size of the reference paths i.e. an oversized participant-generated path results in a scale factor <1. Rotation is the angular offset between the participant-generated and reference paths; less rotation indicates a better match between the produced and reference paths. Shape accuracy was calculated by evaluating the mean distance between corresponding points on the transformed input path and the reference path and, thus, represents how well the participant was able to recreate the qualitative properties of the form irrespective of input scale, location or rotation errors. Lower values represent less ‘error’ and therefore better shape accuracy.

#### 3D tracing

The three-dimensional Cartesian coordinates of the virtual stylus tip and fish were recorded at 50 Hz. These data were filtered using a dual-pass, second-order Butterworth filter with a 5 Hz cut-off frequency. Using these data with the known coordinates of the target trajectory (the path), we computed trial duration (the time it took for a participant to travel from the starting to finishing locations) to evaluate performance.

#### Statistical analyses

First, to examine the overall learning for both the 3D tracing and drawing tasks, we performed paired sample t-tests; an a priori alpha level was set at 0.05. Then, we used regression analyses to examine the contribution of the Beery VMI scores to explaining performance. First, we used a model selection technique (using Mallows' C*p* score [43]) to determine the best model using only a subset of potential predictors. The model selection function performed a search for the best subsets of variables using an efficient branch-and-bound algorithm – it returned Mallows' C*p* scores (where a C*p* score close to or smaller than *p* represents a better fit) for various combinations of predictors. For the 3D tracing task, the potential predictors were: support level (1–8), session (baseline, posttest), repetitions within sessions (1–2), and the three individual Beery scores (VMI, VP, and MC). For the drawing task, the potential predictors were: session (baseline, posttest), scale, rotation, repetitions within sessions (1–3), figure (A–D), and the three individual Beery scores (VMI, VP, and MC). Using the selected subset of predictors, we then performed multiple regression on the dependent measures.

## Results

### DCD-Q and Beery VMI

First we examined scores for the DCD-Q and the Beery VMI to describe the characteristics of population tested. The DCD-Q and the three components of the Beery VMI were distributed across most of the possible ranges for scores, respectively. Thus, some of the scores fell into the range typically associated with Developmental Coordination Disorder (DCD); although in this study, none of the children were diagnosed as DCD. The median scores were: 61 (DCD-Q; with eight children falling under the threshold for “probable” or “suspected” DCD); 25 (VMI); 63 (VP); 23 (MC). Of the children with low DCD-Q scores, four also scored below the 10^th^ percentile on either the VMI or MC from the Beery. Using Shapiro-Wilk, Shapiro-Francis, and Skewness/Kurtosis tests, we tested the distributions for potential departures from normality. None were different from normal (p>0.05 in all cases). We tested the inter-correlations among these scores. The DCD-Q did not correlate well with scores from the Beery. The r-values were as follows: for VMI, r = 0.2; for VP, r = 0.11; and for MC, r = 0.19. Components of the Beery exhibited reasonably good inter-correlation. The r for VMI-VP was 0.34. For VMI-MC, it was 0.56. For VP-MC, it was 0.62.

### 3D tracing

Overall, we found that training in the 3D tracing task yielded significant learning as revealed by a baseline and posttest comparison (t_22_ = 3.3, p<0.01, one-tailed).

### Relation between Beery VMI and 3D tracing

For the 3D tracing task, the potential predictors were: session (baseline, posttest), level of support (1–8), repetitions within sessions (1–2), and the three individual Beery scores (VMI, VP, and MC). Using Mallow's C*p* score we selected a subset of predictors. The model that had the best C*p* score (5.12) contained five predictors: session, support level, and the three individual Beery scores (VMI, VP, and MC).

We then performed a regression on duration using session (with baseline and posttest coded as +1), level of support (coded as 1–8) and the VMI, VP, and MC scores as predictors. We also included the (3) two-way interactions between session and the three Beery scores, and the (3) two-way interactions between support level and the three Beery scores (but not those between the Beery scores), and the (3) three-way session by support level by Beery score interactions as predictors. The overall model was significant (F_(15,720)_ = 33.4, p<0.001) and accounted for 41% of the variance. The significant single factors were session (t_4_ = 2.3, p<0.02) and support level (t_4_ = −8.0, p<0.001). However, there were significant two-way interactions (session by level: t_4_ = −7.3, p<0.01; support level by VP score: t_4_ = −3.4, p<0.001) and one three-way interaction (session by support level by VP score: t_4_ = 3.1, p<0.01).

**Figure 4:**
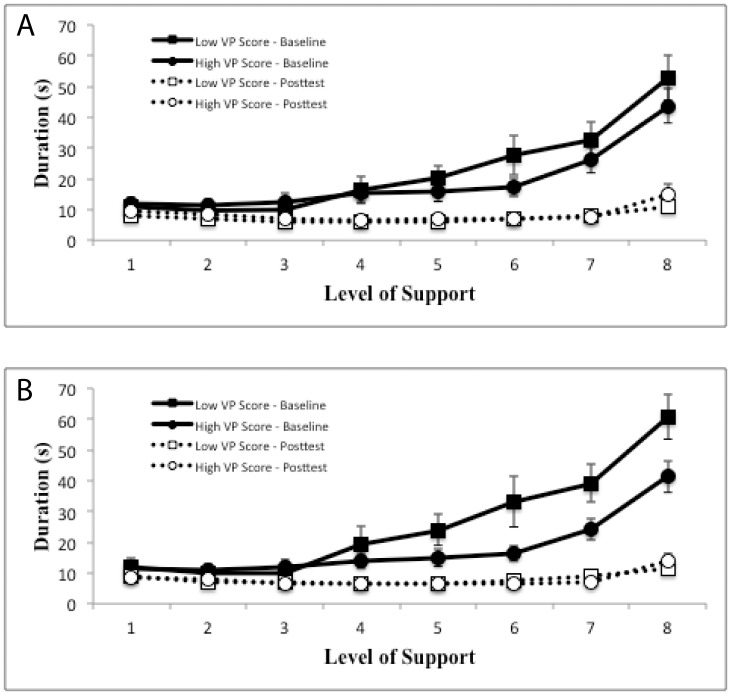
Mean trial durations as function of session, support level, and VP score. A) Mean duration computed by VP scores (high or low) relative to the median score as a function of session (baseline, posttest) and support level (1 =  high, 8 =  low). B) Mean durations computed by VP scores (high or low) relative to the average as a function of session (baseline, posttest) and support level (1 =  high, 8 =  low).

To investigate these effects, we computed means for duration as a function of session (baseline, posttest), support level (1–8), and VP score (high and low, split by the median). These are shown in Figure 4. At baseline, children with higher VP scores were faster at completing the 3D tracing task. At posttest, however, there were no differences between children. This is further reflected in separate analyses for the baseline and posttest data. Using the baseline data with VP and support level as factors, we found that the model accounted for 30% of the variance. Support level was significant (t_2_ = 7.6, p<0.001) as was VP by support level (t_2_ = −2.8, p<0.01). Next, we performed the same analysis on posttest data only. The model only accounted for 3.4% of the variance and none of the factors were significant.

### Drawing

Overall, we found that training in the 3D tracing task yielded improvements in the drawing task with respect to shape accuracy (t_22_ = 2.1, p<0.02, one-tailed). There was no difference between baseline and posttest with respect to scale (t_22_ = −0.2, p>0.5, one-tailed) or rotation (t_22_ = 0.4, p>0.3). The copies, however, tended to be larger than the original (one-sample t-test, with μ = 1: t_45_ = −15.3, p<0.001; 95% confidence interval: 0.76, 0.82) and slightly rotated (one-sample t-test, with μ = 0: t_45_ = 14.6, p<0.001; 95% confidence interval: 4.00, 5.28).

### Relation between Beery VMI and Drawing

For the drawing task, the potential predictors were: session (baseline, posttest), scale, rotation, repetitions within sessions (1–3), figure type (forms A–D), and the three individual Beery scores (VMI, VP, and MC). Using Mallow's C*p* score we selected a subset of predictors. The model that had the best C*p* score (3.39) contained five predictors: session, figure type, scale, rotation, and VP.

**Figure 5:**
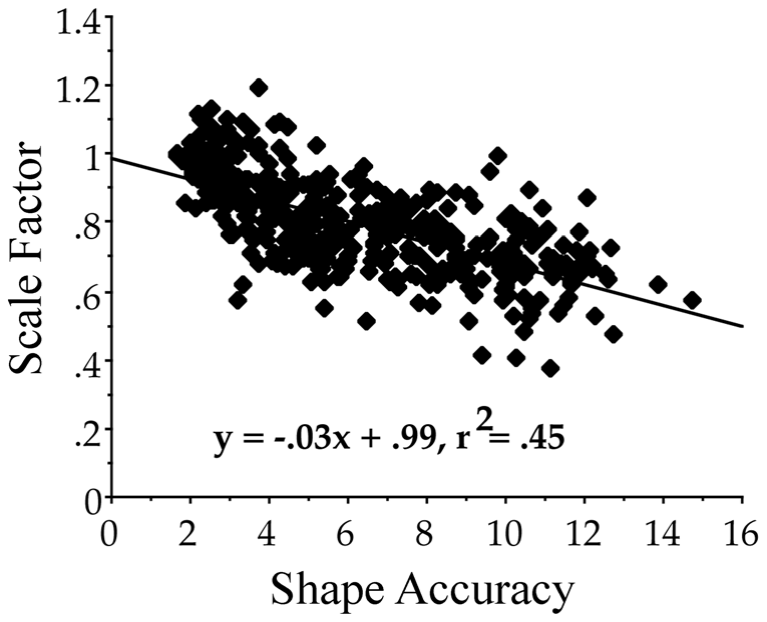
Scale factors plotted as a function of corresponding shape accuracy scores. As the size of the copied forms deviated from the size of the target form, shape accuracy scores worsened.

We performed a regression on shape accuracy using scale factor, rotation, VP score, session (with baseline and posttest coded as – /+1), figure type (coded as 1–4) as predictors. We also included all two-way interactions and three-way interactions involving session as predictors. The overall model was significant (F_(21,530)_ = 19.4, p<0.001) and accounted for 43% of the variance. The significant single factors were session (t_4_ = 2.5, p<0.02), figure type (t_4_ = −3.2, p<0.002), scale factor (t_4_ = −4.8, p<0.001), and VP score (t_4_ = 5.6, p<0.001). However, there were significant two-way interactions (session by scale: t_4_ = −2.6, p<0.01; session by VP score: t_4_ −2.4, p<0.02; figure type by scale: t_4_ = 2.9, p<0.01; figure type by VP score: t_4_ = −2.5, p<0.02; scale by VP score: t_4_ = −5.2, p<0.001) and one three-way interaction (session by scale by VP score: t = 2.5, p<0.02). The figure type effects reflected the fact that one of the figures was easier to copy than the others, namely, the horizontal wave with varying amplitude. Errors decreased over sessions from baseline to posttest. Errors were smaller as the scale factor approached 1, meaning that errors were larger as figures were drawn larger than the targets to be copied. See Figure 5 where the proportional relation between reproduction scale and error is shown. Errors were larger for lower VP scores and smaller for higher VP scores.

**Figure 6:**
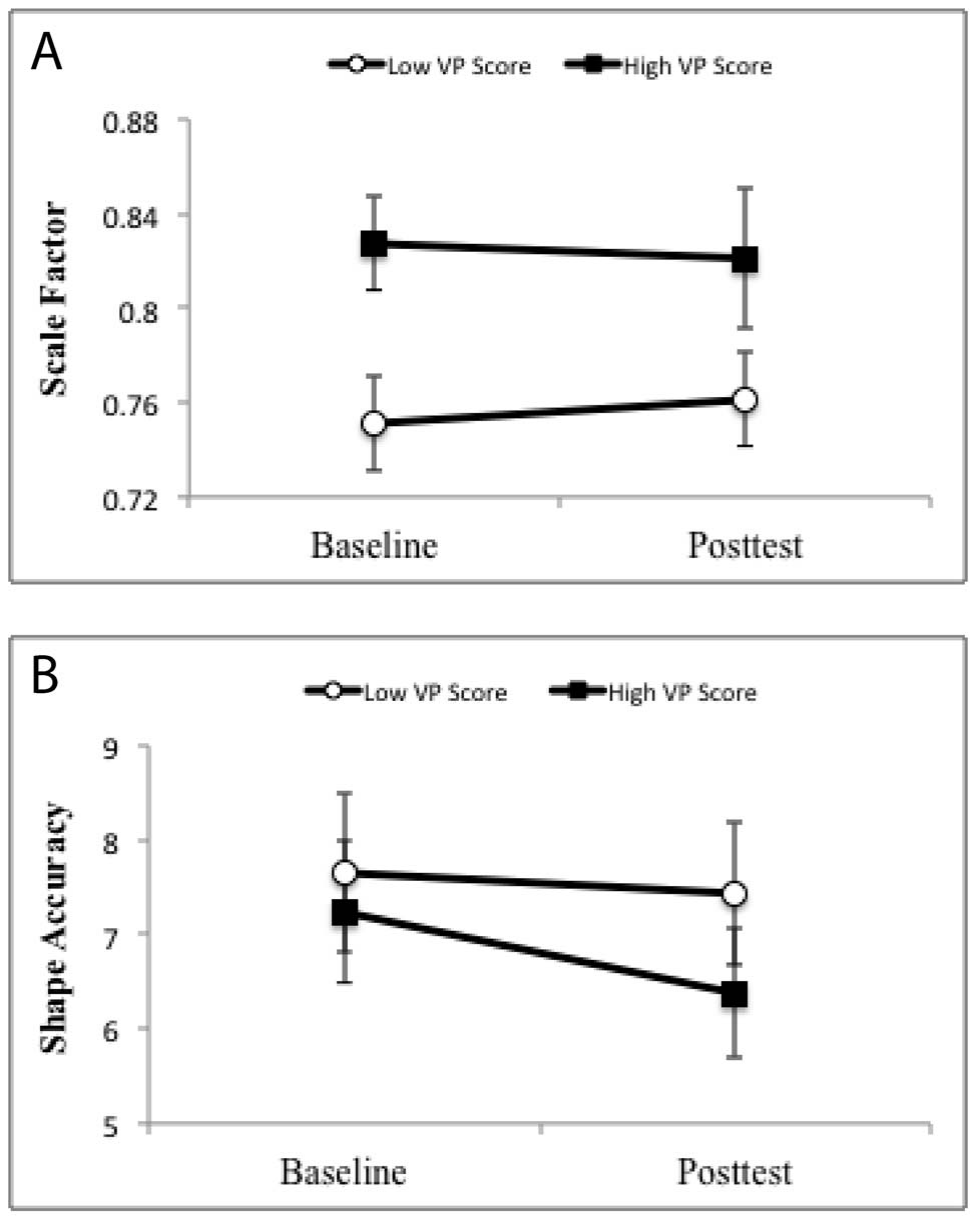
Mean scale factor and shape accuracy as a function of session and VP score. A) Scale factor by VP scores (high or low) relative to the median score as a function of session (baseline, posttest), B) Shape accuracy by VP scores (high or low) relative to the median score at baseline and posttest.

To illustrate these effects, we computed means for scale factor and for shape accuracy as a function of session (baseline, posttest) and VP score (above and below the median). These are shown in Figure 6 (scale factor is shown in Figure 6A, shape accuracy is shown in Figure 6B). For children with high VP scores, scale factors were larger indicating that they were producing shapes closer to the correct scale. Also, children with higher VP scores produced more accurate shapes than children with lower VP scores. With training on the 3D tracing task, scale factors increased and shape accuracy improved (especially for children with higher VP scores). This is further reflected in separate analyses for the baseline and posttest data. Using the baseline data with VP, figure type, and scale factor along with their two-way interactions as factors, we found that the model accounted for 38% of the variance. VP score was significant (t_5_ = 5.197, p<0.001) as was VP by figure type (t_5_ = −2.258, p<0.03) and VP by scale factor (t_5_ = −5.054, p<0.001). Next, we performed the same analysis on posttest data only. The model accounted for 45% of the variance. Again, VP score was significant (t_5_ = 2.294, p<0.03). Additional significant factors were scale factor (t_5_ = −5.800, p<0.001), figure type (t_5_ = −4.215, p<0.001), and the scale factor by figure type interaction (t_5_ = 4.124, p<0.001).

## Discussion

The goal of the present study was to examine the influence of a new training method in a broader context. Previously, Snapp-Childs et al. [1] tested a method for training good manual compliance control in children with Developmental Coordination Disorder (DCD) by comparing baseline-posttest differences in performance by typically developing children and children with DCD. Before training, the task was effectively impossible for the children with DCD to perform without strong support. After training, they could do the task as well as typically developing children. Thus, large differences between the groups were evident in baseline performance but these differences were eliminated in posttest performance. Snapp-Childs et al. [26] further tested the approach used in this training method by comparing learning in groups of participants who either trained passively, by performing a haptic tracking task, or actively, by performing the active 3D tracing task with progressive reduction of support. The results showed far superior learning by the active group as compared to the passive group. In the present study, we investigated the application of this approach to the training of good manual compliance control with young school-aged children and the utility of the Beery VMI in predicting children's performance.

The primary hypotheses tested in this study of young school-aged children were that: 1) Beery scores would predict baseline performance in a 3D tracing task and a drawing task, 2) training of good manual compliance control (first described by Snapp-Childs et al. [1]) would alter any such relationships and 3) training on the 3D tracing task would improve drawing performance. We found evidence supporting all three hypotheses. First, we found that one of the three component scores of the Beery was useful in predicting performance in the 3D tracing and the drawing tasks, but that training altered the utility. That is, for the 3D tracing task, the VP score was useful in predicting performance at baseline, but not at posttest. For the drawing task, the VP score was useful in predicting performance at baseline and posttest. In advance, we had expected the VMI to be the more relevant assessment, especially for the drawing task, simply because it entails the copying of geometric forms. Moreover, previous work has shown that VMI scores are predictive of handwriting quality for children with handwriting problems [44]. However, surprisingly, it was the visual perception (VP) score, not the visual-motor integration (VMI) score or the motor coordination (MC) score that predicted performance. In fact, the VP score predicted the baseline performance on the 3D tracing task in a way that was similar to the classification of participants in our previous study [1] as being typically developing or as having DCD. That is, those with lower VP scores performed substantially worse on the 3D tracing task before training. Also, for the drawing task, the VP score interacted with the scale in that children with higher VP scores produced shapes that were both more similar in size to the target forms and better in shape accuracy. Conversely, children with lower VP scores produced copies that were both less similar in size to the target forms and worse in shape accuracy.

Why did the VP score of the Beery predict the performance in the two tasks, 3D tracking and drawing? The VP task in the Beery requires participants to discriminate subtle differences in complex forms both in respect to the forms themselves and their scale. It tests sensitivity and attention to detail in complex pattern perception. In contrast, the MC task only tests the ability to stay within the lines (i.e. a steady hand). And, while the VMI task tests complex figure copying, it does so with little consideration for scale and more subtle deviations in form production. In respect to the drawing task, our computer-based analyses revealed that the specific scale of the reproduction was important to the successful drawing of the form itself. The perceptual task is essentially the same in the two cases, that is, in the VP subtest of the Beery and our drawing task. In respect to the 3D tracing task, Snapp-Childs et al. [26] showed that visual prospective control is the key to successful skilled performance of this task, that is, visual perception of the torsion and curvature of the path in anticipation of moving the stylus over that path. Thus, good visual discrimination of form and scale is again required and once again, the perceptual ability in the two tasks, the VP Beery task and 3D tracing, is the same. This interpretation is consistent with previous work that has found the association between visual-perceptual abilities and motor skill performance to be task-specific [45].

We also examined whether the training on the 3D tracing task yielded improvements in the drawing task (i.e. was there transfer of learning?). Training in the 3D tracing task yielded improvements in drawing performance. While training improved overall shape accuracy, it also appeared to increase differences between children with low versus high VP scores. This result may be a bit disappointing but is not that surprising. Again, the drawing task demanded (visual) attention to detail in addition to sufficient control of the hand/arm. That is, high visual perceptual ability underpins accurate performance – being able to notice subtle changes in contour and shape is primary for being able to accurately reproduce figures. This interpretation is consistent with the notion that movement stability is a function of perceptual ability [46].

### Future directions

Transfer of learning or the changes in drawing performance, although significant, were small. However, the amount of training at the 3D tracing task was also modest. Much more difficult paths can be tested both during training and posttest. In addition, an earlier study [1] included a second parameter in addition to the level of support (attraction), namely, amount of friction along the path. Higher levels of this friction were found to cause unskilled participants to come off the path. Thus, continued and extensive training in compliance control is available through use of this additional parameter, especially when coupled with increased complexity of path shape, and likely this would yield larger changes in drawing performance. In future, we will also investigate differences in effective training using 3D as compared to 2D paths lying in a horizontal plane as well as the generalization of these changes to “pen-and-paper” writing.

## Conclusions

In sum, this approach to training is showing good promise as a means to help children improve in performance of fine motor manual tasks like drawing or potentially, handwriting, and the results indicate that the methods might be well applied within the public schools.
